# What can we learn from the hair of the dog? Complex effects of endogenous and exogenous stressors on canine hair cortisol

**DOI:** 10.1371/journal.pone.0216000

**Published:** 2019-05-22

**Authors:** Rowena M. A. Packer, Alexander M. Davies, Holger A. Volk, Holly L. Puckett, Sarah L. Hobbs, Robert C. Fowkes

**Affiliations:** 1 Clinical Science and Services, Royal Veterinary College, Hawkshead Lane, North Mymms, Hertfordshire, United Kingdom; 2 Endocrine Signalling Group, Royal Veterinary College, Royal College Street, London, United Kingdom; University of Modena and Reggio Emilia, ITALY

## Abstract

Hair is an emerging biological matrix in which to measure chronic HPA axis activity, offering a longer term view into an animal’s life. We explored effects of exogenous (e.g. lifestyle, medications, social environment) and endogenous (e.g. disease, behaviour) stressors on hair cortisol concentration (HCC) in a population of Border Collies (BCs). Owners of BCs were recruited and reported their dog’s lifestyle, clinical history, anxiety-related behaviour, and collected a white hair sample from their dog’s dorsal neck region. HCC was determined using established methods with a commercial cortisol assay kit. Samples from 135 BCs were analysed, with 91 healthy controls and 44 diagnosed with epilepsy as a model disease. Factors associated with higher HCC included psychosocial stressors (living with three or more other dogs) and lifestyle (engaging in competitive flyball); while factors associated with lower HCC included anxiety (stranger-directed and non-social), health (epilepsy diagnosis, with number of seizures to date negatively correlated with HCC) and medication (certain anti-epileptic drugs were associated with elevated or reduced HCC). These novel results highlight the potential of chronic stress with frequent or persisting HPA-axis hyperactivity leading to a state of hypocortisolism, and the need to consider stressor recency and recurrence when interpreting HCC data.

## Introduction

The domestic dog is a species of global importance, with an estimated 10.5 million kept as companion animals in the UK alone [1]. Other key canine roles include military and police dogs, assistance dogs for a variety of medical conditions, with the dog also considered as a model for many heritable human diseases [2]. The welfare of dogs in all of these roles is of societal interest, and quantifying their stress levels has been a topic of scientific interest over the past half a century. Stressors may be actual or perceived, and can be psychological or physiological in origin [3]. Stress in dogs is commonly studied using behavioural observations in combination with measurement of cortisol, an endogenous glucocorticoid hormone found in biological matrices including blood, urine and faeces, to quantify hypothalamic-pituitary-adrenal axis (HPA) activity. A multitude of endogenous and exogenous stressors have been identified to alter cortisol levels in dogs including unpredictable aversive stressors (e.g. sound blasts, electric shocks and falling bags [4], working [5], hunting [6], thunderstorms [7], and dog park visits [8]). Understanding the effects of potential stressors on stress physiology in dogs is vital for evidence-based optimization of their care, particularly where stressors are modifiable or preventable.

As a non-invasive measure, salivary cortisol has received great attention, with a recent meta-analysis of samples from 1205 dogs revealing significant effects of signalment (sex, neuter status, age), husbandry (regular living environment) and sampling methods (time in environment before testing, testing environment, owner presence during testing, and collection media) on salivary cortisol levels [9]. Although valuable in measuring the effects of real-time acute stressors, a number of factors limit salivary cortisol’s use in stress measurement. Salivary cortisol follows a circadian rhythm in dogs making comparisons between samples between different times of day imprecise [10], may require stimulants to facilitate sufficient sample collection [11], and may be sensitive to the sample collection environment and method [9].

While the short-term stress response is adaptive and crucial in facilitating the body’s ability to cope in emerging situations [12, 13], overexposure to glucocorticoids due to chronic hyperactivation of the HPA axis in chronic stress can have detrimental effects on the body and result in endocrine, metabolic, autoimmune or psychiatric disorders [14]. There is a need for more chronic measures of stress in the dog that do not require repetitive sampling regimes to measure HPA activity over days, weeks or month. This may include quantifying effects of chronic disease, behavioural disorders and the environment in which dogs are housed. To meet this need, hair cortisol concentrations (HCC) are increasingly being measured in dogs, considered a promising indicator of long term HPA-axis activity. A core benefit of measuring HCC is its potential to provide a long term endocrine profile over the duration of hair growth, insensitive to the impact of acute stress [15]. Hair sampling is inexpensive and non-invasive, can be performed easily and rapidly by the dog’s carer or an experimenter and is very easily preserved pre-analysis. Positive correlations between HCC and salivary [16] and fecal [15] cortisol levels have already been established in dogs. Despite canine HCC being in its relative infancy compared with salivary cortisol [9], a variety of preliminary associations between HCC and stressors have been identified, including health status [17, 18], behaviour [19, 20], social environment [21, 22] and lifestyle [19].

The aim of this study was to explore the effects of exogenous (e.g. lifestyle, social environment, medications) and endogenous (e.g. disease, behaviour) stressors on HCC in a single breed of dog, the Border Collie (BC). BCs are a popular breed internationally, used in a variety of roles including working, companion and competition, and commonly studied in canine behaviour and cognition research [23, 24]. As studies of the relationship between chronic disease using heterogeneous populations of diseased animals have not found significant results to date, idiopathic epilepsy (IE) has been chosen as the single disease of interest in this study. IE is a common chronic neurological disorder in dogs, characterised by recurrent seizure activity, and estimated to affect 0.6-.075% of dogs [25, 26]. The BC breed is predisposed to a severe form of IE [27]. Seizures have previously been demonstrated to be associated with acute spikes in salivary cortisol in dogs [28]; however the long term effects are not yet understood, despite seizures being recurrent in this chronic condition.

## Methods

### Study design

To explore these relationships, we drew on a sample of BCs including both healthy dogs and dogs diagnosed with IE. Hair samples were collected by their carers in the home environment to maximise the number of participants while reducing potential stress of collection by an unfamiliar person. Alongside hair samples, owners submitted an online questionnaire capturing relevant data on their dog’s lifestyle, behaviour and clinical history. All protocols were approved by the Royal Veterinary College Ethics and Welfare Committee URN BSC320177 and all experiments were performed in accordance with relevant guidelines and regulations. All owners consented to their involvement in the study via a consent statement in the online questionnaire.

### Recruitment of participants and inclusion criteria

Dogs were recruited via advertising the study on social media, including BC breed clubs and carer support groups for dogs with IE, on Twitter, and via the The Kennel Club through their BARC site and direct messages to registered BC owners. Dogs were not recruited based on their behaviour to avoid biasing the sample towards those dogs perceived by their owners to have behaviour problems, with the study marketed as ‘*The Great Big Hairy Border Collie Study*’. Dogs were eligible for inclusion in the healthy control group if they had no history of chronic disease, or any acute health problems in the three months preceding sample collection. Dogs were eligible for inclusion in this IE group if they had met the criteria for Tier I diagnostic certainty, as described by the International Veterinary Epilepsy Task Force (IVEFT) [29]. This includes a history of two or more unprovoked epileptic seizures occurring at least 24 h apart, age at epileptic seizure onset of between six months and six years, unremarkable inter-ictal physical and neurological examination, and no significant abnormalities on minimum data base blood tests and urinalysis.

Owners first completed the online questionnaire which included their contact details. Owners of eligible dogs were then electronically sent a sample collection instruction sheet, and instructed to send hair samples to the study center by post. On arrival, samples were matched to their questionnaire records by dog name and date of birth.

### Hair collection, storage, and processing

Building upon the findings of previous studies, this study was designed to use only white hair from the neck region of BCs, with all samples collected by owners within a one-month period in July 2017 to account for potential effects of season on HCC. Owners were provided with a sample collection instructions sheet to improve consistency of sampling. Hair samples were obtained by cutting ~0.5g of hair with a pair of scissors as close to the skin as possible, from the dorsal neck region. Owners were requested to send the sample in a sealed plastic bag in an envelope via post.

On arrival at the study center the samples were re-bagged and labelled with the dog’s study ID, and stored in a refrigerator at −20°C until the day of the assay. Cortisol levels in the samples were determined with a commercial salivary cortisol assay kit and performed according to methods described previously (Salimetrics, Newmarket, UK; [30, 31]). Briefly, samples were first finely cut into short sections to prevent tangling during pulverization and weighed. The entire shaft of the hair sample was included in the analyses, without separation of guard hair and wool. Samples were then powdered using a ball mill and 5mm stainless steel ball bearing, at 30Hz for 10 minutes. Samples were then immersed in 1.5 mL of absolute methanol (18 h at 20°C) before centrifugation (3,000 × G for 5 min at 4 C). The supernatants were dried (18 h at 65°C) before resuspension in the assay buffer supplied in the kit and absorbance measurement at 450 nm. Values were expressed as micrograms of cortisol per deciliter normalized per milligram hair sample. The intra-assay coefficient of variance was 6.7% and inter-assay coefficient of variance was 11.0%.

### Questionnaire

Alongside hair sampling, carers completed a questionnaire hosted on the online platform SurveyMonkey from July–August 2017 (S1 File). Section (1) consisted of general questions regarding the owner (e.g. location, gender, age) and their dog (e.g. age, sex, neuter status, weight). Section (2) included more detailed questions on the dog’s provenance and lifestyle, including their role (pet, working, sporting), social environment (people and other dogs in the household) and involvement in leisure activities. Training techniques used by owners was taken from [32] and categorized into reward-based only, punishment-based only, or a combination of both. Section (3) included the questions from the five anxiety-related sections of the Canine Behaviour and Research Questionnaire (C-BARQ): non-social fear, dog-directed fear, stranger-directed fear, pain sensitivity and separation anxiety [33]. Section (4) documented current and historical health problems: owners reported any health problems their dog had been diagnosed with and whether they were (a) currently affected; (b) now resolved, but affected within the past three months, or (c) now resolved, and had not been affected within the past three months for the purposes of excluding dogs in groups (a) and (b). Owners reported any medication or surgeries their dog had undergone in the past three months. Section (5) screened for IE diagnosis in all dogs, including key questions to ascertain tier I certainty for IE diagnosis according to International Veterinary Epilepsy Task Force (IVETF) criteria, including number of seizures experienced, age at first seizure and results of any diagnostic tests (blood/urinalysis). Finally, section (6), which was only presented to those dogs meeting inclusion criteria for the IE group, explored IE phenotype (e.g. seizure frequency/per month, history of cluster seizures and/or status epilepticus) and treatment history.

### Data handling and statistics

All analyses of were carried out in SPSS Statistics 24 (Armonk, NY: IBM Corp). Due to a HCC not conforming to the normal distribution, non-parametric statistical analyses were performed. Bivariate spearman’s rank correlations were used to examine the relationship between HCC and continuous variables including age, weight and C-BARQ anxiety scores. The Mann-Whitney or Kruskall-Wallis tests were used to examine effects of categorical effects on HCC e.g. lifestyle and disease variables depending on the number of categories in a variable. In all tests p<0.05 was considered to be significant and data was presented as mean (± SD) for normally distributed data, or median (interquartile range [IQR]) for non-normally distributed data.

## Results

### Demographics

Samples with completed surveys meeting inclusion criteria were available for 135 dogs (S2 File). The majority of respondents were from England (71.9%), followed by Scotland (8.1%) and Wales (6.7%). Owners were predominantly female (90.4%) and most commonly aged 46–60 years old (46.7%). Half of dogs were registered with the Kennel Club (51.1%). Over half of dogs were male and the majority were neutered (39.3% male neutered, 20.0% male entire, 30.4% female neutered, 10.4% female entire); no effect of sex or neuter status on HCC were found (p>0.05). The mean age (months) of study dogs was 59.1 ± 33.2 and the mean weight (kg) of was 19.1kg ± 3.54; no association between age or weight and HCC was observed (p>0.05).

### Lifestyle

**Fig 1:**
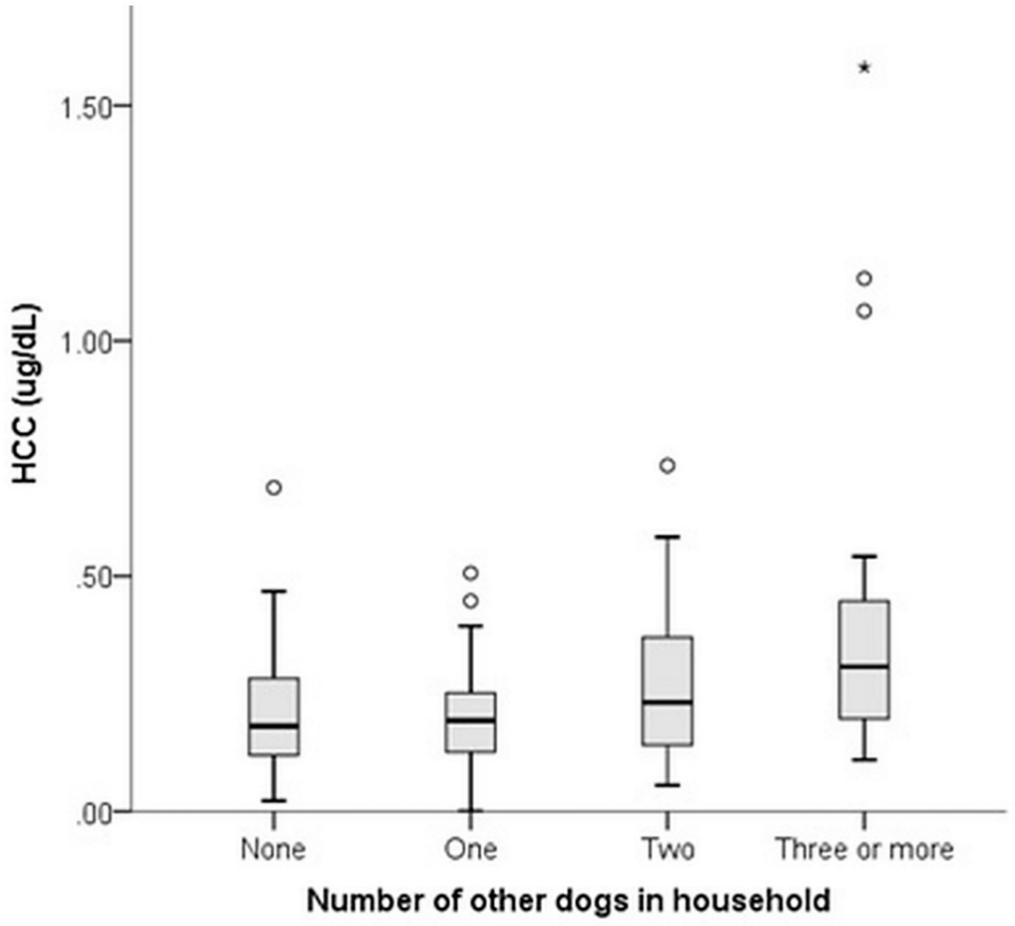
Relationship between HCC and number of other dogs in the household in a population of 135 Border Collies.

Over half of dogs were acquired from a breeder (57.1%: pet breeder 18.0%, working breeder 33.1%, show breeder 6.0%), with 20.3% acquired from farms, 10.5% from a family member or friend and a minority rehomed (12.1%). Lifestyle roles included companion (56.3%), working (39.3%) and competition dogs (4.4%); with no effect of role on HCC (p = 0.19). Dogs lived with a mean number of 2.17 ± 1.26 people, with no association between HCC and number of people in the household (r = -0.14, p = 0.259). Dogs were left alone without people for a mean of 3.10 hours ± 2.29 per day on an average weekday, and there was no association between length of time left alone and HCC (r = 0.09, p = 0.36). Dogs lived with a mean number of 1.55 ± 1.47 other dogs, with a significant relationship between HCC and number of other dogs in the household (KW = 10.62, p = 0.014): dogs that lived with three or more other dogs had significantly higher HCC than dogs that lived with only one dog (p = 0.018), or no other dogs (p = 0.042) (Fig 1).

**Fig 2:**
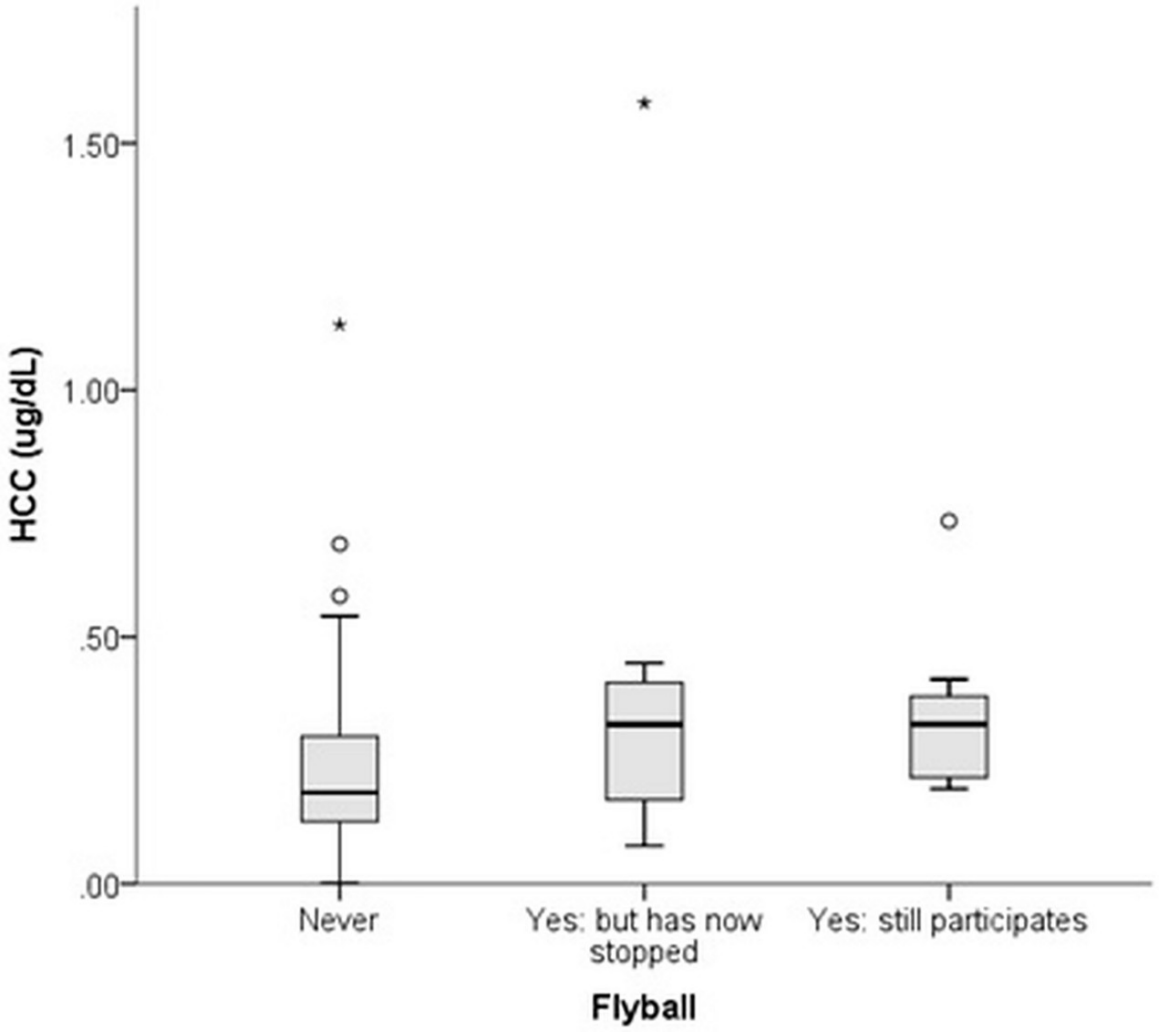
Relationship between participation in flyball and HCC in a population of 135 Border Collies.

The most common activity dogs were involved in was agility, followed by flyball, with a minority involved in dog showing (Table 1). Dogs that currently participate in flyball exhibited higher HCC than those that had never participated (Fig 2).

**Table 1:** Canine activities the study population involved in (n = 135 Border Collies). Percentage of dogs involved in each activity stated with median (IQR) HCC.

Activity	Currently participates		Used to but now stopped		Never participated		KW	p
	%	HCC	%	HCC	%	HCC		
Agility	40.6%	0.26 (0.17–0.35)	18.0%	0.15 (0.10–0.27)	41.4%	0.19 (0.14–0.32)	5.45	0.066
Flyball	6.8%	0.32 (0.19–0.41)	9.4%	0.32 (0.14–0.41)	83.8%	0.18 (0.12–0.30)	7.64	0.022
Dog showing	2.5%	0.32 (0.19–0.33)	5.1%	0.16 (0.10–0.34)	92.4%	0.19 (0.13–0.31)	1.18	0.554

Two thirds of owners used a combination of both punishment and reward-based training techniques to train their dog (64.2%), with the remaining 35.8% using solely reward based methods, and no owners reporting that they used solely punishment-based methods. No effect of training method was found upon HCC (M-W = 1510.5, p = 0.952).

### Epilepsy sub-population

**Fig 3:**
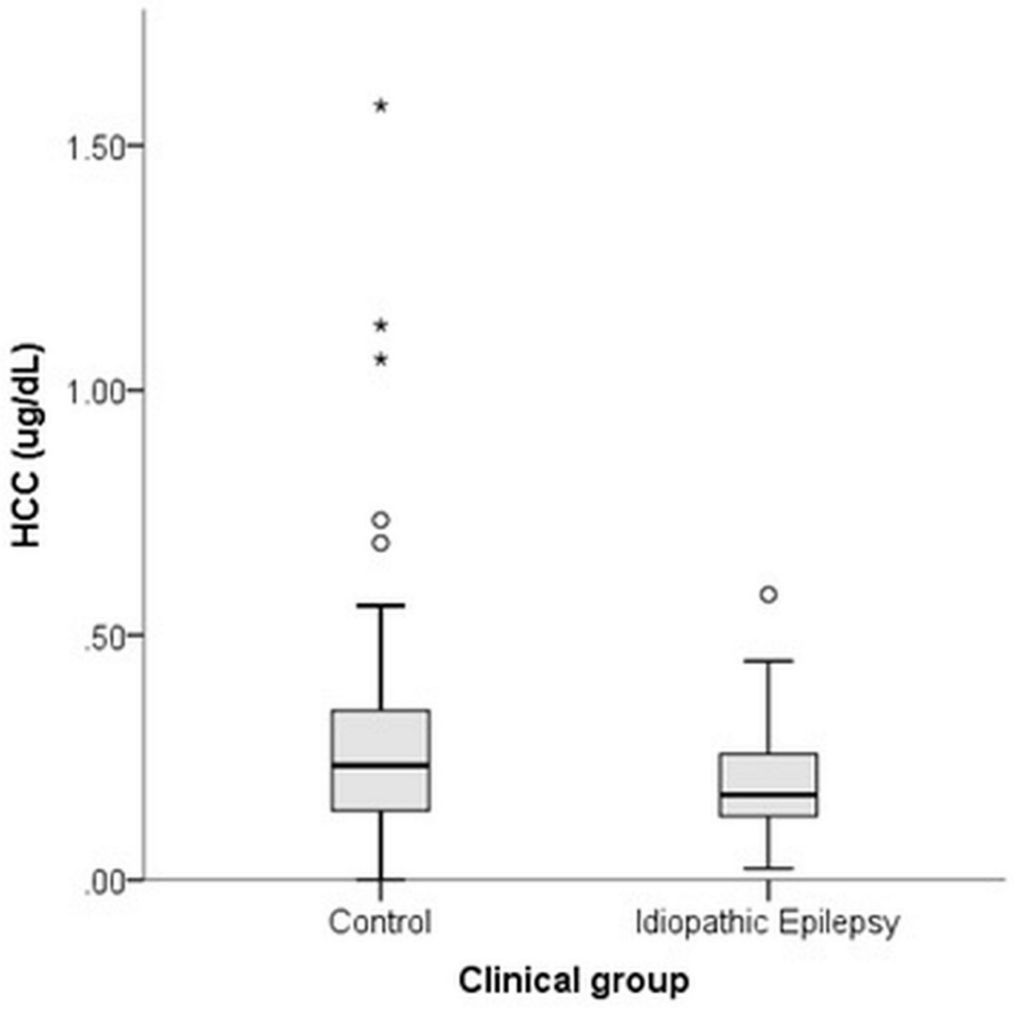
Difference in HCC between Border Collies with epilepsy (n = 44) and healthy controls (n = 91).

Forty-four dogs met IVETF tier I criteria for diagnosis of IE, with 38.6% (n = 17) having additionally undergone MRI of the brain, and 34.1% (n = 15) cerebrospinal fluid analysis to confirm their diagnosis. Dogs with IE had significantly lower HCC than controls (M-W = 4.33, p = 0.037) (Fig 3).

**Fig 4:**
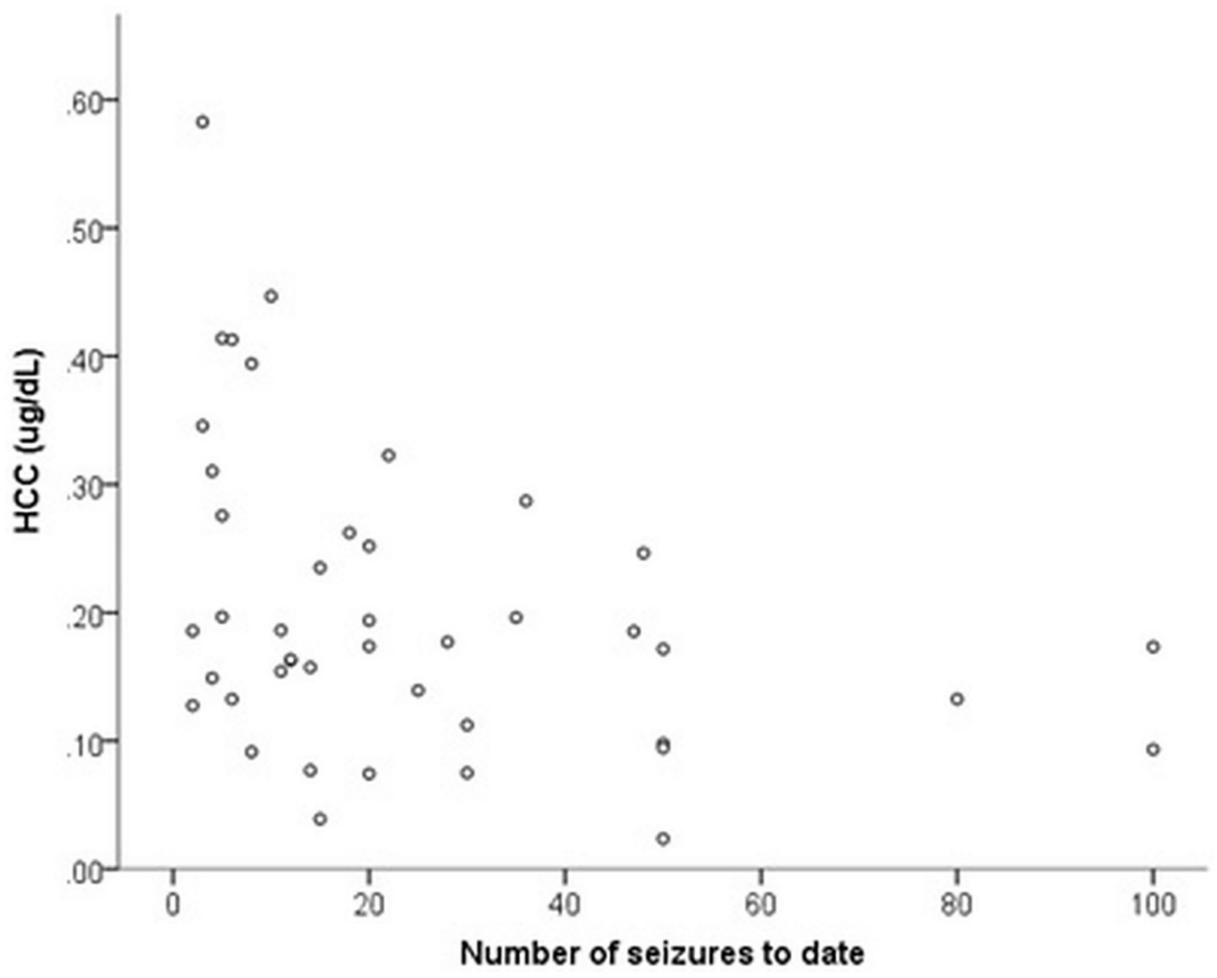
Relationship between HCC and number of seizures experienced to date in a population of 44 Border Collies diagnosed with idiopathic epilepsy.

The mean age (months) of epileptic dogs was 66.6 months ± 31.3 and mean weight (kg) was 20.4kg ± 3.6. The majority of dogs had experienced cluster seizures (81.8%, n = 36) while a minority had experienced status epilepticus (27.3%, n = 12); neither were associated with HCC (p>0.05). The mean duration of IE (time since diagnosis) was 32.6 months ± 28.7, and mean number of seizures experienced to date was 24.5 ± 24.4. Number of seizures to date was significantly negatively correlated with HCC (-0.38, p = 0.011) (Fig 4), while duration of IE was not (-0.26, p = 0.088).

All dogs with IE were being treated with at least one anti-epileptic drug (AED), and over half of dogs received two or more AEDs (52.3%, n = 23). The most common AEDs used were phenobarbital (75.0%, n = 33), potassium bromide (36.4%, n = 16), levetiracetam (34.1%, n = 15) and imepitoin (11.4%, n = 5). Effects of AEDs upon HCC were identified; HCC was significantly lower in dogs treated with PB or KBR, but significantly higher in dogs treated with IMP (Table 2).

**Table 2:** Effects of anti-epileptic drugs upon HCC in 44 Border Collies diagnosed with IE. HCC is stated as median (IQR).

Anti-epileptic drug	HCC Median (IQR)				Statistical comparison	
	n	Yes	n	No	M-W U	p
Phenobarbital (PB)	33	0.17 (0.07–0.22)	7	0.39 (0.28–0.45)	36.00	0.003
Potassium bromide (KBR)	16	0.16 (0.11–0.22)	24	0.22 (0.16–0.34)	85.50	0.003
Imepitoin (IMP)	5	0.35 (0.21–0.52)	35	0.17 (0.10–0.25)	148.00	0.011
Levetiracetam (LEV)	15	0.17 (0.10–0.28)	25	0.19 (0.12–0.27)	165.50	0.543

### Anxiety profiles

**Fig 5:**
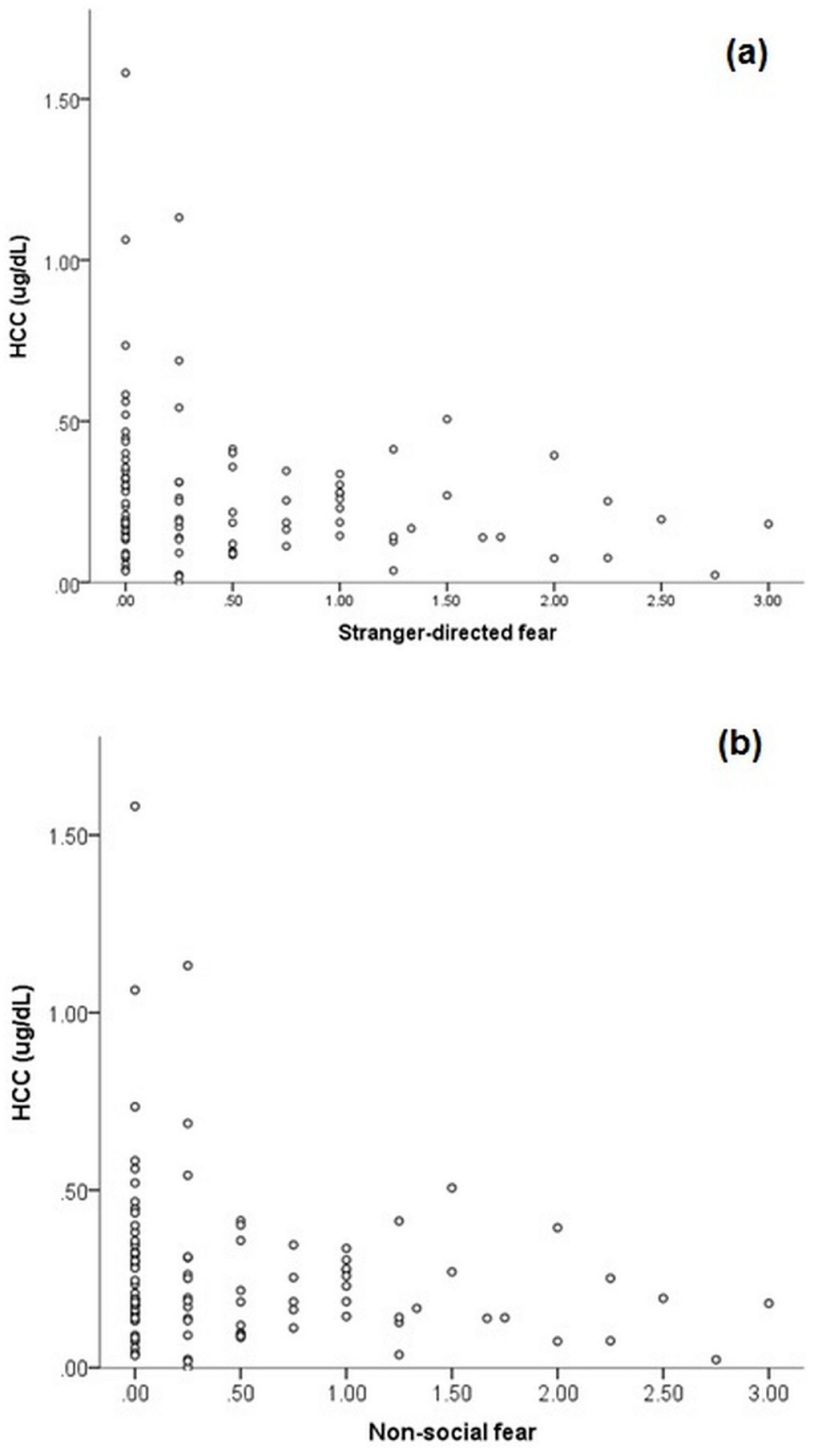
Relationship between HCC and (a) stranger-directed and (b) non-social fear in a population of 135 Border Collies.

No difference in C-BARQ anxiety scores was identified between epileptic and control dogs (p>0.05); however, due to overall differences in HCC identified between epileptic and control dogs, anxiety scores were considered for both all samples combined and for control dogs separately. Negative correlations were identified between HCC and the C-BARQ score “non-social fear” (Table 3) in both the full sample and when controls were analyzed separately, and “stranger-directed fear” in control dogs only (Fig 5).

**Table 3:** Relationship between HCC and C-BARQ anxiety traits in n = 135 Border Collies.

C-BARQ domain	Group Mean ± SD			Correlation with HCC (all dogs, n = 135)		Correlation with HCC (controls only, n = 91)	
	Overall	Epilepsy	Control	r	p	r	p
Non-social fear	0.82 ± 0.64	0.79 ± 0.59	0.83 ± 0.67	-0.21	0.029	-0.30	0.011
Dog-directed fear	0.83 ± 0.88	0.69 ± 0.84	0.89 ± 0.89	-0.12	0.214	-0.19	0.149
Stranger-directed fear	0.45 ± 0.67	0.45 ± 0.65	0.46 ± 0.69	-0.16	0.089	-0.31	0.012
Pain sensitivity	0.67 ± 0.68	0.57 ± 0.53	0.71 ± 0.74	-0.14	0.172	-0.17	0.187
Separation anxiety	0.20 ± 0.30	0.21 ± 0.26	0.19 ± 0.32	-0.02	0.828	-0.05	0.684

## Discussion

To our knowledge this is the largest and most in-depth single-breed study of the effects of endogenous and exogenous stressors on HCC in the domestic dog. The results highlight complex effects of stressors on the HPA axis, including the social environment, leisure activities, disease, medication, and behavioural problems, with both up and downregulation of the HPA axis observed. These data corroborate the results of previous investigations into HCC into dogs, including potentially negative effects of a large number of social companions in the home [16] and involvement in competitive sports [19], while identifying novel effects on the impact of anxiety and chronic neurological disease on HCC.

Potential sources of psychosocial stress were identified in the study population, with dogs living alone or with one companion dog exhibiting lower HCC than dogs living with three or more other dogs. These findings mirror previous findings of the social environment in dogs; in one study, dogs living alone had significantly lower HCC than dogs from multi-dog households [16], and in a further study, thunderstorm-phobic dogs living alone had higher baseline salivary cortisol before a simulated thunderstorm than those living in multi-dog households [7]. These results suggest that co-habiting with other dogs may be a cause of day-today chronic stress; however, other studies have found apparently positive effects of social companions; for example, HCC reduced in dogs following a move from solitary to paired housing [21] and length of time spent alone was positively correlated with HCC in dogs in a single-dog household, but not a multi-dog household [22]. Greater examination of dog-dog interactions within households are required to better characterize relationship quality between conspecifics and determine whether elevations in HCC in multi-dog households is due to greater exposure to negatively valenced sources of arousal (e.g. competition over resources, antagonistic interactions), or positively valenced sources of arousal (e.g. enhanced availability of play and positive social interaction) which may both conceivably induce HPA responses in dogs.

As previously reported, different lifestyles and activities may affect canine HPA axis activity, with competition dogs exhibiting higher HCC than companion or working dogs [19]. Although no difference was found in the HCC between dogs classed by owners as companion, working or competition in the present study, dogs involved in flyball, a competitive dog sport, had higher HCC than those never involved in this sport. Flyball is a high energy team racing sport which involves high levels of arousal during competitions, with dogs potentially exposed to high levels of frustration while waiting their turn to sprint for the ball and exposed to other highly aroused dogs in this environment. As dog sports are popular within the BC breed, it is possible that certain behavioural traits that predispose dogs in this breed to high performance in flyball also predispose those individuals to higher HCC; indeed, HCC has previously been positively correlated with ‘reactivity’ in dogs [20].

Competition dogs have previously been found to have higher HCC than companion and working dogs, particularly in January, while training less during the ‘out of competition’ season [19]. This effect was previously interpreted as a consequence of the unpredictability of a competition dog’s schedule, with rest days interspersed with intensive training sessions; this contrast may be a potential stressor for dogs. In comparison, professional working dogs (who may maintain relatively high activity throughout the day) and companion dogs (who may only engage in light training sessions) may have a more consistent and predictable lifestyle. Repeat sampling of HCC during on and off season periods combined with acute measures of cortisol on competition and non-competition days and could confirm this effect with flyball. With competitive dog sports increasing in popularity internationally, their impact on canine stress should be considered.

Two behavioural variables, non-social fear and stranger-directed fear were found to be negatively associated with HCC in BCs. Dogs showing greater signs of fear/anxiety towards non-social stimuli (e.g. loud noises, traffic) and dogs showing greater signs of fear/anxiety towards strangers exhibited lower HCC. Although it may be hypothesised that dogs most sensitive to these stressors would exhibit higher HCC, as chronic stressors with potentially unpredictable exposure, it is possible that the HPA axis of these dogs may have become dysregulated following chronic exposure, leading to a state of HPA hypoactivity, or ‘vital exhaustion’ [34, 35]. Previous studies using acute measures of stress have identified potential HPA hypoactivity associated with chronically stressful environments; in a recent meta-analysis of canine salivary cortisol studies, dogs living in shelters exhibited significantly lower salivary cortisol than those living in the home or working dog kennels [9]. Although initially considered an unexpected finding by those authors, it was considered likely that these relatively low levels of cortisol in an environment perceived to be aversive to dogs (e.g. unfamiliarity, exposure to unknown dogs and humans) is likely due to exhaustion dysregulation of the HPA axis. This phenomena is observed in chronically stressed humans when they no longer feel able to cope with chronic life stressors, and is reportedly accompanied by feelings of increased irritability and demoralisation [35]. Recent studies have found similar results in human anxiety; generalised anxiety disorder (GAD) is characterized by excessive worry and anxiety regarding a variety of life problems, and indeed GAD patients reported significantly higher perceived stress scores than controls [36]. However, GAD patients exhibit lower HCC compared to age, gender and lifestyle-matched controls [36]. This hypocortisolemia suggests a downregulation of the HPA axis with chronic anxiety, and is seen with other psychiatric disorders; for example major depression (MD); where MD patients exhibit lower HCC compared to controls [37]. At present, there is no canine reference range available to determine whether HCC values fall within a ‘normal’ range and may reflect pathological hypoactivity; further studies with a greater number of hair samples across breeds are required to achieve this.

Previous studies of the effects of disease upon HCC have produced conflicting results; two studies identified elevated HCC in diseased dogs compared to healthy controls (dogs with atopic dermatitis [17] and Cushing’s disease (hypercortisolism) [18] have higher HCC than healthy dogs); however, two studies of dogs with chronic disease (heterogeneous groups including osteoarthritis, cardiac failure, liver disease, neoplasia and diabetes) did not identify differences between diseased dogs and healthy controls [18, 22]. Despite this previous lack of difference, effects of epilepsy, chronic seizure activity and AED treatment were identified in the current study, which may highlight the need for studying defined populations of diseased animals.

There is a complex relationship between stress and epilepsy, with stress and subsequent cortisol release potentially acting as a trigger for seizures [38, 39], and seizures themselves acting as a stressor upon the body. Spontaneous seizure activity in dogs with IE has previously been found to have a marked effect upon HPA activity; increases in salivary cortisol post-seizure are substantial (median 531% at 20 minutes; 265% at 40 minutes), with significant differences between time-matched non seizure and post-seizure samples at 40 minutes post-seizure [28]. To the authors’ knowledge, HCC has only been used to study epilepsy once to date in humans. In a study of children presenting with their first epileptic seizure, higher HCC was found in these patients (sampled within 24 hours of their first seizure) compared to controls, which may indicate that HPA hyperactivity may be part of the initial epileptogenic process [40]. This sampling point avoided acute HPA effects induced by seizure activity. Whether HCC is initially elevated in dogs with IE is currently unknown but warrants investigation. In the current study, dogs with established IE exhibited lower HCC, with increasing number of seizures associated with decreasing levels of HCC. This novel finding may represent HPA dysregulation in epilepsy patients; with seizures representing a recurrent unpredictable stressor upon the body, followed by a potentially mentally aversive post-ictal phase which may last minutes to hours. It is already established that the HPA axis may be attenuated by seizures when measuring cortisol in other biological matrices; reduced inhibitory control of the HPA axis is seen in epilepsy patients, as manifested by a prolonged increase of serum cortisol levels and a slowed return to baseline levels after exposure to a stressor [41, 42]. In addition, recent studies have identified reduced steroidogenesis in girls with mutations in the protocadherin 19 (PCDH19) gene, known to cause female-limited epilepsy (PCDH19-FE). In this population, cortisol levels (as measured from blood samples) were lower compared to age-matched controls, along with other neuroactive steroids including allopregnalanone, pregnenolone sulfate, 17OH-progesterone, progesterone, and cortisol [43]. The authors speculated that attainment of a normal adrenocortical function could be a therapeutic goal in this condition, to reduce seizure susceptibility to environmental challenges as a result of reduced cortisol production capacity.

The findings of the present study are complicated by the effects of AEDs; in the current study, all dogs with IE in were drug-treated, with two AED’s were found to reduce HCC (phenobarbital and potassium bromide), while one increased HCC (imepitoin). In humans, cortisol secretion is similar in temporal lobe epileptic patients and pseudoseizure patients receiving AEDs, suggesting a direct effect of the drugs upon the adrenal cortex.[44]. In dogs, the effects of phenobarbital on adrenocortical function have been investigated, with some reporting effects of phenobarbital upon results of ACTH stimulation and dexamethasone suppression test [45], while others report no effect [46, 47]. Further investigation of the effects of AEDs on the HPA axis are required to interpret these results more fully, including the effects of different dosages which was not explored here. In addition, studies of drug-naïve canine patients, longitudinally studied from seizure onset would be of value, and may offer insights into relationship between epileptogenesis and seizure activity and the HPA axis in isolation.

Although not yet utilized to the same extent as salivary cortisol (where 61 peer-reviewed studies were included in a meta-analysis between 1992–2012 [9]), as an emerging technique in the quantification of chronic stress in dogs, efforts should be made to optimise and harmonise methods used to process and analyse hair samples to allow for comparison between studies. Although studies of Labrador Retrievers and German Shepherd dogs have demonstrated no difference in HCC in old and new-growth hair samples [16], a potential limitation of this study was the use of old hair rather than a shave-reshave protocol to obtain new hair growth. There is likely significant variation in hair growth rates across breeds [16], which were overcome by using a single-breed study design. It is possible that within-breed individual differences may have led to the hair sample representing different timeframes for different dogs in this study. In humans, approximately 1 cm/month is generally accepted as the hair growth rate [48], and future studies should quantify hair growth and HCC differences across and within breeds in dogs. Sampling effects have also been identified that should be considered in study design; HCC has been found to be higher in January than in May and September [19], and HCC in yellow or white hair has been found to be lower than in black hair [16, 49]. As such, time of year and sample hair colour should be controlled for in study design or accounted for in analyses. Finally, the timing and duration of stressors may also be an important consideration for HCC studies e.g. when the stressors occurred prior to sampling if an ‘event’, and their duration prior to sampling if a ‘status’, such as those variables identified here such as health status or social environment.

## Conclusions

Hair cortisol is a promising measure of chronic stress in dogs, influenced by both endogenous and exogenous stressors, including behaviour, disease, medication, lifestyle and the social environment. Stressors may cause both up or down regulation of HCC and thus caution should be taken in interpreting results, with characteristics of the stressor considered including recency and recurrence. HCC may provide new insights to support or challenge mechanistic notions of chronic stress responses in dogs, and highlights the need to expand this body of research in dogs.

## Supporting information

S1 File Online survey questions. (PDF)

S2 File Data file. (XLSX)
