# Supplementary material for: What can we learn from the hair of the dog? Complex effects of endogenous and exogenous stressors on canine hair cortisol
Source: PLoS One. 2019 May 22;14(5):e0216000. doi: 10.1371/journal.pone.0216000 (PMC6530888; doi:10.1371/journal.pone.0216000)
Supplement: S1 File — (PDF) [file pone.0216000.s001.pdf]

Royal Veterinary College
University of London

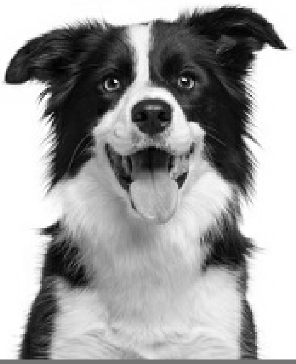

The Great Big Hairy Border Collie Study

Introduction to survey

Do you own a Border Collie and want to help fight epilepsy in your breed?

What is this study about?

Epilepsy is the most common neurological disorder affecting dogs. We understand that epilepsy can be a very stressful condition for owners to witness and manage. With the Border Collie being one of the **hardest breeds to treat** with anti-epileptic medication, and **cluster seizures** common in the breed, we are committed to carrying out research to gain a **greater understanding of epilepsy in Border Collies**, to make life better for both dogs and their owners.

Stress is increasingly being recognised as an important part of epilepsy in both people and animals. This works in two directions:

- **Seizures may lead to stress**, with our previous work showing that the stress hormone 'cortisol' increases after a seizure
- **Stress may lead to seizures** in dogs with epilepsy, with some dogs having stress 'triggers' (e.g. a visit to the vets, a thunderstorm) that result in a seizure

We are interested in exploring stress in Border Collies with epilepsy, not just immediately after a seizure, but their **long term stress levels.** We can measure stress hormones in your dog's **hair**, with 'cortisol' deposited in your dog's hair as it grows, depending on their stress levels at that time.

In this study we will investigate whether Border Collies **with epilepsy** have different levels of stress hormones in their hair compared to Border Collies **without epilepsy,** and whether other elements of their lifestyle affect these levels. Within the epilepsy group, we will explore **factors associated with high or low stress levels.** If stress is found to be an important influence on seizures, then we hope to investigate stress-reduction therapies as a seizure management method in the future.

Who can get involved?

We would like to include **Border Collies diagnosed with epilepsy** <u>**AND**</u> **Border Collies without epilepsy.**
All owners must be over 18 years old.
ALL dogs must be:
- Over 6 months old
- Must have been owned you for at least 3 months
- Must have had no short term health conditions in the past 3 months

Please check your dog fits the criteria of one of these groups:

Epilepsy group	Control group
Experienced at least 2 seizures that were over 24 hours apart	No history of seizures
First seizure aged between 6 months and 6 years of age	Must not have received any steroid treatment in the last 3 months
No abnormalities on blood and urine tests	Must have no diagnosed endocrine disorders e.g. Cushing's disease, hypothyroidism, hyperthyroidism
Diagnosed by a vet **at least 3 months ago**	

What do we want from you?

To take part in this study, we require you to:
- Complete the following **survey**
- Send us a **sample of your dog's hair** (full instructions will be given at the end of the survey)

* 1. The data collected in this study will be collated and stored at the Royal Veterinary College in London (RVC). Your data will be anonymised as appropriate, and your contact details will be used only to contact you if we need clarification of the answers to any questions forming part of this questionnaire. Your personal information will be held and used in accordance with the Data Protection Act 1998 and will not be disclosed to any unauthorised person or body. Only the study investigators (Dr Rowena Packer, Prof Holger Volk, Dr Rob Fowkes, Mr Alex Davies) will have access to the data collected as part of this study. The anonymised results of this study may be used in scientific publications. We are happy to send you an electronic summary of the study results (by email only) at the end of the study

If you have any queries regarding during the study, please contact Dr Rowena Packer (rpacker@rvc.ac.uk) or Mr Alex Davies (adavies6@rvc.ac.uk)

Do you confirm that you are over 18 years old and consent to take part in this study?

- ◯ Yes
- ◯ No

Royal Veterinary College
University of London

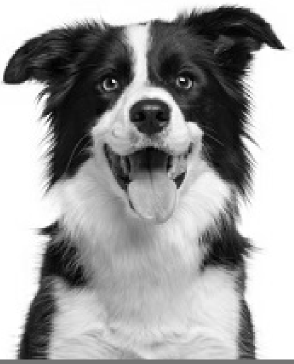

The Great Big Hairy Border Collie Study

This survey and you

We would like a couple of details about you and how you were led to completing this survey.

2. In which country do you live?

3. Are you the main carer of your dog? (i.e. you are the main person in your household to feed your dog, administer medication etc.)

- ◯ Yes
- ◯ No
- ◯ I don't know

4. Please select your gender

- ◯ Male
- ◯ Female
- ◯ Prefer not to disclose

5. Please select your age range (in years)

- ◯ 18-30
- ◯ 31-45
- ◯ 46-60
- ◯ 61-75
- ◯ over 76
- ◯ Prefer not to disclose

RVC Royal Veterinary College University of London

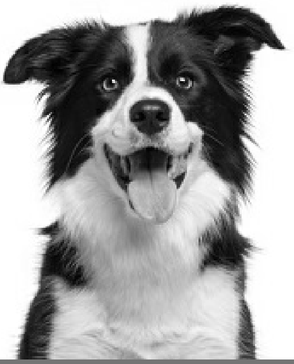

The Great Big Hairy Border Collie Study

Your dog

* 6. What is your dog's name?
(N.B. we require this information to identify your dog and match it to it's hair sample)

* 7. What is your surname?
(N.B. we require this information to identify your dog and match it to it's hair sample)

8. What sex is your dog

- ○ Male Neutered
- ○ Female Neutered
- ○ Male Entire
- ○ Female Entire

9. Is your dog registered with the Kennel Club?

- ○ Yes
- ○ No
- ○ I don't know

10. What is the weight of your dog in kilograms (kg)?

11. How old is your dog?
Please state in MONTHS (e.g. if 5 years 5 months, enter 65)
N.B. If unknown, please estimate

12. If known, state your dogs date of birth

Date of birth:

DD/MM/YYYY

13. How old was your dog when you acquired them?

Please state in MONTHS

14. Where did you acquire your dog from?

- Breeder (Primarily working dogs)
- Breeder (Primarily pet dogs)
- Breeder (Primarily show dogs)
- Rescue centre (e.g. RSPCA, Dogs Trust, Blue Cross)
- Border Collie breed rescue
- Family or friend
- Pet shop
- Farm

15. Is there a known history of epilepsy in your dogs family/line?

- Yes
- No
- I don't know

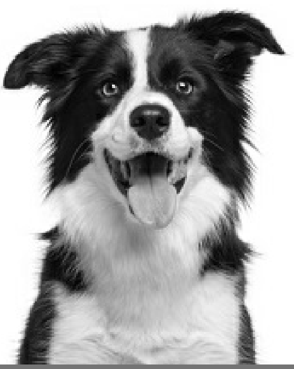

The Great Big Hairy Border Collie Study

Your dog's lifestyle

We will now ask you some questions about your dog's lifestyle

16. In which category would you class your Border Collie?

- ◯ Pet
- ◯ Working
- ◯ Competition
- ◯ Other (please specify)

17. How many people does your dog live with?

	Number
Children under 5 years old	
Children aged 5-10 years old	
Children aged 11-16 years old	
Children aged 17-18 years old	
Adults aged over 18 years old	

18. How many other animals does your dog live with?

	Number
Dogs	
Cats	
Rabbits	
Rodents (e.g. guinea pigs, rats, mice, hamsters, gerbils)	
Birds	
Fish, amphibians and reptiles	

19. How many hours is your dog usually left alone without people on an averageweekday?
Please enter as a number

RVC Royal Veterinary College University of London

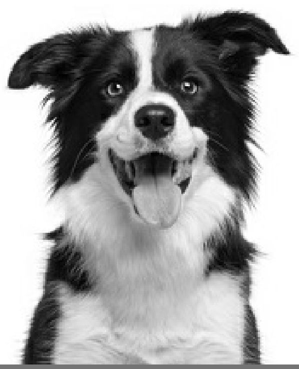

The Great Big Hairy Border Collie Study

Training

We will now ask you some brief questions about your dog's training and behaviour

20. Has your dog taken part in any of the following activities?

	Never	Yes: but has now stopped	Yes: still participates
Agility	○	○	○
Dog showing	○	○	○
Flyball	○	○	○

21. Which of the following training techniques have you ever used with your dog? (tick all that apply)

- ☐ Food rewards (giving a treat) when the dog does a correct behaviour
- ☐ Bark activated citronella collar (automatically sprays strong smelling liquid to stop barking)
- ☐ Harness to prevent pulling on lead
- ☐ Verbal punishment (e.g. telling off or shouting) when the dog does something wrong
- ☐ Shutting away (physically removing from the room, sometimes called 'time out') when the dog behaves badly
- ☐ Stroking or patting when the dog behaves well, verbal praise
- ☐ Pet corrector (aerosol type spray directed at dog to interrupt unwanted behaviour)
- ☐ Electronic boundary fence to prevent the dog from wandering off the property
- ☐ Physical punishment (e.g. smacking) when the dog does something wrong
- ☐ Withhold treats or food when the dog does something wrong
- ☐ Ignoring (stopping giving the dog any attention when he or she does something wrong)
- ☐ Electronic training collar (to give an electronic correction when the dog does something wrong)
- ☐ Choke chain (metal collar that a tightens on dogs neck) to prevent pulling on lead
- ☐ Playing (e.g. throwing a toy when the dog does a correct behaviour)
- ☐ Physical manipulation (e.g. pushing the bottom down) to encourage correct behaviour
- ☐ Pulling back on lead when the dog pulls
- ☐ Bark activated electronic training collar (automatically gives electronic correction to stop barking)
- ☐ Water pistol (sprayed to interrupt a behaviour when dog does something wrong)
- ☐ "Husher" device that prevents the dog barking
- ☐ Clicker training (using the 'click' sound, followed by a treat when the dog does a correct behaviour)
- ☐ Stopping forward movement or changing direction when the dog pulls on the lead
- ☐ Non-verbal sound distraction (e.g. can of stones, 'training discs', or air horn) to stop the behaviour when the dog does something wrong
- ☐ Prong collar (metal chain with extensions that put pressure on dog's neck when it pulls on the lead)

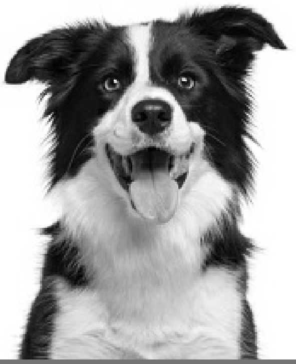

Your dog's behaviour

We will now ask you a variety of questions about how your dog behaves in different situation, and their general personality. We are interested in whether dogs who behave differently have different stress levels.

22. Dogs sometimes show signs of anxiety or fear when exposed to particular sounds, objects, persons or situations. Typical signs of mild to moderate fear include: avoiding eye contact, avoidance of the feared object, crouching or cringing with tail lowered or tucked between the legs, whimpering and whining, freezing, and shaking and trembling. Extreme fear is characterised by exaggerated cowering, and/or vigorous attempts to escape, retreat or hide from the feared object, person or situation.

By clicking on the following scales, please indicate your own dog's recent tendency to display fearful behaviour in each of the following contexts:

	No signs of fear or anxiety	Mild fear/anxiety	Moderate fear/anxiety	Severe fear/anxiety	Extreme fear/anxiety	Not applicable
Toward unfamiliar dog visiting the home						
When approached directly by an unfamiliar dog of the same or larger size						
When approached directly by an unfamiliar dog of a smaller size						
When approached directly by an unfamiliar male adult while away from the home						
When approached directly by an unfamiliar female adult while away from the home						
When approached directly by an unfamiliar child while away from the home						
When unfamiliar persons visit the home						
In response to sudden or loud noises (e.g. vacuum cleaner, car backfire, road drills, objects being dropped, etc.)						
In heavy traffic						
In response to strange or unfamiliar objects on or near the pavement (e.g. plastic trash bags, leaves, litter, flags flapping, etc.)						
During thunderstorms, firework displays, or similar events.						
When first exposed to unfamiliar situations (e.g. first car trip, first time in elevator, first visit to veterinarian, etc.)						
In response to wind or wind-blown objects						
When examined or treated by a veterinarian						
When having its claws clipped by a household member						
When groomed or bathed by a household member						

23. Some dogs show signs of anxiety or abnormal behaviour when left alone, even for relatively short periods of time. Thinking back over the recent past, how often has your dog shown each of the following signs of separation-related behaviour when left, or about to be left, on its own:

	Never	Seldom	Sometimes	Usually	Always	Not applicable
Shaking, shivering, or trembling when left or about to be left on its own	○	○	○	○	○	○
Excessive salivation when left or about to be left on its own	○	○	○	○	○	○
Restlessness, agitation, or pacing when left or about to be left on its own	○	○	○	○	○	○
Whining when left or about to be left on its own	○	○	○	○	○	○
Barking when left or about to be left on its own	○	○	○	○	○	○
Howling when left or about to be left on its own	○	○	○	○	○	○
Chewing or scratching at doors, floor, windows, and curtains when left or about to be left on its own	○	○	○	○	○	○
Loss of appetite when left or about to be left on its own	○	○	○	○	○	○

RVC Royal Veterinary College University of London

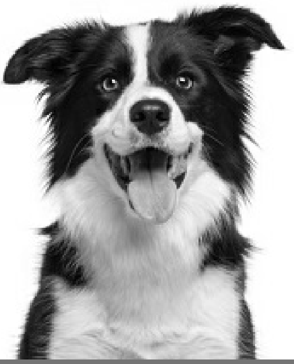

The Great Big Hairy Border Collie Study

Your dog's health

* 24. Has your dog experienced any of the following health problems? (tick all that apply)

	No - Never affected	Yes - Currently affected	Yes - Now resolved/treated, but my dog was **affected in the past 3 months**	Yes - Now, resolved/treated and my dog has been **unaffected in the past 3 months**
Hip problems	○	○	○	○
Arthritis	○	○	○	○
Dental problems	○	○	○	○
Colitis	○	○	○	○
Bladder problems	○	○	○	○
Anal sac problems	○	○	○	○
Knee problems	○	○	○	○
Spinal problems	○	○	○	○
Cancer	○	○	○	○
Elbow problems	○	○	○	○

* 25. Is your dog affected by affected by any long-term health conditions (other than epilepsy or those listed above)?

○ No

○ Yes (please specify)

26. How many times has your dog been to the vet in the past three months?

* 27. Has your dog been treated with any prescribed medication in the past 3 months?
(N.B. other than routine worming/vaccinations, or anti-epileptic drugs used to control your dog's epilepsy, if your dog is affected by epilepsy)?

- ◯ No
- ◯ Yes (please specify)

Please state one medication per line

28. Has your dog had any surgery in the past 3 months?

- ◯ No
- ◯ Yes (please specify)

Royal Veterinary College University of London

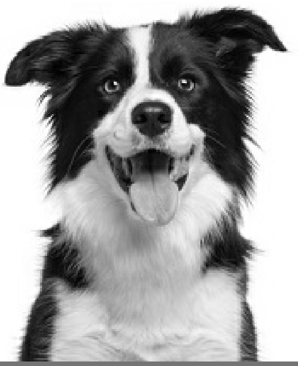

The Great Big Hairy Border Collie Study

Epilepsy screening questions

We will now ask some questions about your dog's medical history to decide which study group they belong to

* 29. Has your dog ever had a seizure?

- ◯ Yes
- ◯ No
- ◯ I don't know

* 30. Has your dog been diagnosed with epilepsy by a vet?

- ◯ Yes
- ◯ No

RVC Royal Veterinary College University of London

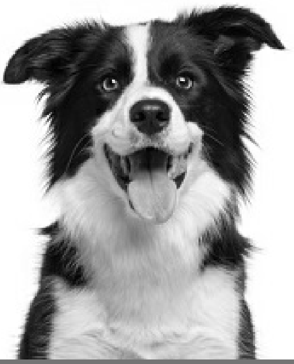

The Great Big Hairy Border Collie Study

Epilepsy screening

* 31. To your knowledge, has your dog had 2 or more seizures?
N.B. if there have only been 2, were they more than 24 hours apart?

- ◯ Yes
- ◯ No

* 32. Was your dog's first seizure aged between 6 months and 6 years of age?

- ◯ Yes
- ◯ No

* 33. Has a vet carried out blood and urine tests on your dog and found no identifiable causes for his/her seizures?

- ◯ Yes - and no cause was found
- ◯ Yes - and a cause was found
- ◯ No - these tests were not carried out

RVC Royal Veterinary College University of London

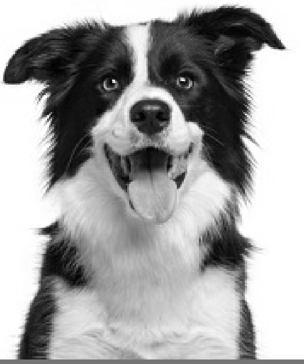

The Great Big Hairy Border Collie Study

Your dog's epilepsy

34. How old was your dog when they had their first seizure? (in months)
N.B. If not known, state "Unknown"

[ ]

35. How many seizures has your dog had in total? (please estimate if precise number has not been recorded)
N.B. If not known, state "Unknown"

[ ]

36. Which vet did you see to discuss your dog's seizures?

- ○ My local vet(s) only
- ○ My local vet(s) AND a Neurology Specialist

* 37. What tests were carried out by your vet? Tick all that apply.

- ☐ Blood tests
- ☐ Urine tests
- ☐ MRI of the brain
- ☐ CT of the brain
- ☐ Lumbar puncture / Cerebrospinal fluid (CSF) analysis
- ☐ Tests were done, but I am uncertain of the details

Other (please specify)

[ ]

* 38. What is the diagnosis for your dog's seizures?

- [ ] Idiopathic epilepsy (i.e. no cause found)
- [ ] Other cause e.g. a brain tumour, liver problems, a stroke, meningitis

Please note the cause if not listed above:

39. When did your dog last have a seizure?

- ○ Within the last 24 hours
- ○ Within the 7 days
- ○ 8-14 days
- ○ 15-21 days
- ○ 22-28 days
- ○ 1-3 months ago
- ○ 4-6 months ago
- ○ 7-12 months ago
- ○ Over 12 months ago
- ○ I don't know

* 40. Approximately how many seizures has your dog had in the past 3 months?

41. If known, please provide us with the dates of your dog's seizures over the past 6 months
Please provide the date in DD/MM/YY format along with number of seizures
e.g. 02/05/17 x1, 22/05/17 x 2

42. Approximately how long do your dog's seizures last on average?

- ○ Under 1 minute
- ○ 1 minute - 2 minutes
- ○ 2 minutes - 3 minutes
- ○ 3 minutes - 4 minutes
- ○ 4 minutes - 5 minutes
- ○ Over 5 minutes

* 43. Has your dog ever have more than one seizure in one day (24 hour period)?

- ○ No - Only one seizure has occurred within 24 hours
- ○ Yes - More than one seizure has occurred within 24 hours
- ○ I don't know

* 44. Has your dog ever experienced a seizure that lasted for longer than 5 minutes?

- Yes
- No
- I don't know

* 45. Has your dog ever had two or more seizures in a row without fully regaining consciousness in between them?

- Yes
- No
- I don't know

Royal Veterinary College
University of London

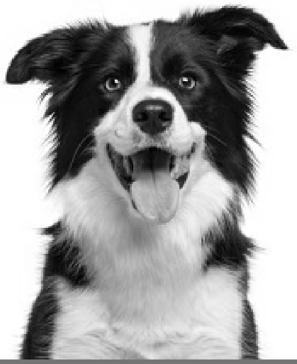

The Great Big Hairy Border Collie Study

Your dog's treatment

* 46. Does your dog currently receive any of the following epilepsy medications?

- [ ] No anti-epileptic medication
- [ ] Imepitoin (Pexion)
- [ ] Phenobarbital (Epiphen, Phenoleptil)
- [ ] Potassium Bromide (Epilease, Libromide)
- [ ] Levetiracetam (Keppra)
- [ ] Zonisamide
- [ ] Gabapentin
- [ ] Pregablin
- [ ] Chlorazepate
- [ ] Felbamate
- [ ] Phenytoin
- [ ] Tiagabine
- [ ] Vigabatrin

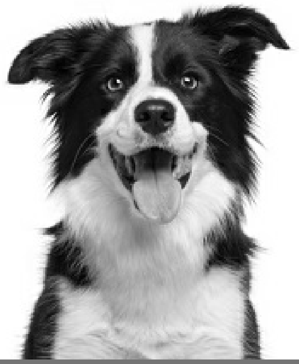

The Great Big Hairy Border Collie Study

Thank you and data information

Thank you very much for your time and effort in supporting this investigation of epilepsy in Border Collies.

Now you have finished the survey, we hope you will be able to provide us with a hair sample from your dog:

Instructions on how to collect hair samples are available on our Facebook page https://www.facebook.com/rvccanineepilepsyresearch/

If you would prefer an electronic copy, please email Alex on adavies6@rvc.ac.uk

Please send your dog's hair sample to us by **Monday 14th August** for it to be included in our analyses

47. To be able to send you instructions for how to collect and send a hair sample to us, we require your email address. Please provide your preferred email address below:

Email
